# Wissen für gesunde Lebenswelten: Eine Datenbank zum Praxistransfer von Erkenntnissen aus systematischen Übersichtsarbeiten

**DOI:** 10.1007/s00103-021-03309-w

**Published:** 2021-04-27

**Authors:** Adrienne F. G Alayli, Christine Witte, Wolfgang Haß, Hajo Zeeb, Thomas L. Heise, Jens Hupfeld

**Affiliations:** 1 Abteilung 5 Unterstützung der Krankenkassen bei Leistungen zur Gesundheitsförderung und Prävention in Lebenswelten, Bundeszentrale für gesundheitliche Aufklärung (BZgA), Maarweg 149–161, 50825 Köln, Deutschland; 2 Referat Prävention, GKV-Spitzenverband, Berlin, Deutschland; 3 Leibniz-Institut für Präventionsforschung und Epidemiologie – BIPS, Bremen, Deutschland; 4 Health Sciences Bremen, Universität Bremen, Bremen, Deutschland

**Keywords:** Wissenschafts-Praxis-Transfer, Lebensweltansatz, Gesetzliche Krankenkassen, Rapid Review, Scoping Review, Wirksamkeit, Knowledge translation, Setting approach, Health insurance funds, Rapid review, Scoping review, Effectiveness

## Abstract

Die Datenbank „Wissen für gesunde Lebenswelten“ wurde durch das GKV-Bündnis für Gesundheit, eine gemeinsame Initiative der gesetzlichen Krankenversicherung (GKV) zur Weiterentwicklung und Umsetzung von Gesundheitsförderung und Prävention in Lebenswelten, entwickelt. Sie soll Krankenkassen und weitere Fachkräfte und Beteiligte in der Praxis bei der Planung und Umsetzung evidenzbasierter Maßnahmen der Gesundheitsförderung und Prävention in Lebenswelten unterstützen. In Ergänzung zu bestehenden Interventionsdatenbanken stellt sie Erkenntnisse aus systematischen Übersichtsarbeiten zur Verfügung. Ziel dieses Beitrags ist es, die Datenbank vorzustellen, Nutzungsmöglichkeiten zu beschreiben sowie Weiterentwicklungsmöglichkeiten zu diskutieren.

Die Datenbank beinhaltet Erkenntnisse zu Wirksamkeit und Umsetzungsstrategien lebensweltbezogener Maßnahmen der Gesundheitsförderung und Prävention. Sie enthält strukturierte Zusammenfassungen von 3 Arten Übersichtsarbeiten: systematische Reviews, Scoping-Reviews und Rapid-Reviews. Die Datenbankeinträge (aktuell *n* = 13) können mit verschiedenen Suchfunktionen (u. a. Freitextsuche, Schlagwortsuche und Suchfilter) recherchiert werden. Die Erstellung der Datenbankeinträge erfolgt qualitätsgesichert mittels eines standardisierten Formulars und im Vieraugenprinzip. Zentrale Informationen sind kurz dargestellt und Fachbegriffe erläutert, um einen niedrigschwelligen Zugang zu ermöglichen. Expertinnen und Experten der gesetzlichen Krankenkassen werden fortlaufend in den Entwicklungsprozess der Datenbank eingebunden.

Im Rahmen der Weiterentwicklung ist geplant, die Datenbank um Erkenntnisse aus neuen Übersichtsarbeiten des GKV-Bündnisses für Gesundheit sowie aus weiteren Quellen zu ergänzen. Zudem werden zukünftig Qualitätsbewertungen der eingeschlossenen Übersichtsarbeiten dargestellt und begleitende Maßnahmen zur Förderung des Wissenschafts-Praxis-Transfers entwickelt.

## Abstract (en)

The database “Insights for healthy settings” was developed by the *GKV-Bündnis für Gesundheit*, a joint initiative of all health insurance funds for developing and implementing setting-based health promotion and prevention measures. It aims to support health insurance funds and other practice partners in planning and implementing evidence-based health promotion and prevention measures in different settings. The database complements existing intervention databases by providing insights based on findings from systematic reviews. This article aims to present the database, describe possible applications, and discuss options for further development.

The database contains findings on the effectiveness and strategies for implementation of setting-based health promotion and prevention measures. In addition to structured summaries of systematic reviews, it includes summaries of scoping reviews and rapid reviews. Different search options (e.g., free text search, subject headings, and search filters) can be used to find database entries (currently *n* = 13). Quality-assured database entries are created by means of a standardized form and based on the four-eyes principle. To allow for easy access, key findings are presented briefly, and technical terms are clarified. Experts from health insurance funds are continuously involved in the development process of the database.

As part of the database’s further development, new reviews of the *GKV-Bündnis für Gesundheit* as well as from other sources will be added. Additionally, quality assessments of included reviews will be depicted and supporting activities developed to further encourage translation of scientific knowledge into practice.

## Einleitung

Rahmenbedingungen und Strukturen in den Lebenswelten spielen eine zentrale Rolle für die Gesundheit und das Gesundheitsverhalten der Bevölkerung. Fußgängerfreundliche Infrastrukturen in Stadtbezirken können bei deren Bewohnerschaft beispielsweise das Risiko für Adipositas, Diabetes mellitus Typ II und Bluthochdruck reduzieren [1]. Auch die Verfügbarkeit gesunder Nahrungsmittel in Schulen kann ein gesünderes Ernährungsverhalten bei Schulkindern fördern [2]. Präventionsmaßnahmen haben daher ein höheres Erfolgspotenzial, wenn sie Maßnahmen der Verhaltensprävention mit einer gesundheitsförderlichen Gestaltung von Lebenswelten verknüpfen. Seit der Ottawa-Charta der Weltgesundheitsorganisation von 1986 hat dieser auch als Lebenswelt- oder Settingansatz bezeichnete Ansatz international an Bedeutung gewonnen [3]. In Deutschland wurde der Lebensweltansatz 1989 mit der Einführung des §20 im SGB V, dem Sozialgesetzbuch für die Gesetzliche Krankenversicherung (GKV), erstmals für die Krankenkassen gesetzlich verankert und von ihnen insbesondere in der betrieblichen Gesundheitsförderung (BGF) entwickelt. Mit dem Gesetz zur Stärkung der Gesundheitsförderung und der Prävention (Präventionsgesetz – PrävG) im Jahr 2015 wurde der Lebensweltansatz im SGB V auf andere Lebenswelten übertragen und deutlich gestärkt.

Im Zuge der Umsetzung des PrävG hat sich das GKV-Bündnis für Gesundheit als gemeinsame Initiative aller gesetzlichen Krankenkassen zur Weiterentwicklung und Umsetzung von Gesundheitsförderung und Prävention in Lebenswelten gebildet. Das Bündnis fördert u. a. Strukturaufbau und Vernetzungsprozesse, die Entwicklung und Erprobung gesundheitsfördernder Konzepte für sozial und gesundheitlich benachteiligte Zielgruppen sowie Maßnahmen zur Qualitätssicherung und wissenschaftlichen Evaluation. Die Aufgaben des GKV-Bündnisses für Gesundheit setzt die Bundeszentrale für gesundheitliche Aufklärung (BZgA) mit Mitteln der gesetzlichen Krankenkassen im Auftrag des GKV-Spitzenverbandes gemäß §20a Abs. 3 und 4 SGB V um.

Das GKV-Bündnis für Gesundheit leistet auch Beiträge zu einer stärkeren Evidenzbasierung lebensweltbezogener Maßnahmen der Gesundheitsförderung und Prävention. Im Rahmen des Bündnisses werden Synthesen bestehender Evidenzquellen erstellt, zum Beispiel durch systematische Übersichtsarbeiten zu nationalen und internationalen Studien. Zudem werden Bestandsaufnahmen bestehender gesundheitsfördernder Angebote durchgeführt sowie Handlungsempfehlungen für Fachkräfte und Beteiligte in der Praxis entwickelt. Die Zahl der methodisch überzeugenden Studien im Bereich der lebensweltbezogenen Gesundheitsförderung und Prävention aus Deutschland ist im internationalen Vergleich noch gering und die Evidenz uneinheitlich [4]. Vor diesem Hintergrund fördert das GKV-Bündnis für Gesundheit gezielt anwendungsbezogene Forschungsvorhaben und unterstützt die Krankenkassen bei Evaluationsvorhaben, um Evidenz zu Konzepten der lebensweltbezogenen Gesundheitsförderung zu schaffen. Damit evidenzbasierte Ansätze häufiger in die praktische Umsetzung kommen, ist das GKV-Bündnis für Gesundheit bestrebt, die vorhandene Evidenz für Fachkräfte und Beteiligte in der Praxis leicht zugänglich zu machen. Dazu werden insbesondere Übersichtsarbeiten auf der Informationsplattform www.gkv-buendnis.de veröffentlicht. In Workshops mit Expertinnen und Experten der Krankenkassen sowie weiteren Akteurinnen und Akteuren werden Ergebnisse ferner hinsichtlich ihrer Anwendungsmöglichkeiten reflektiert. Um wissenschaftliche Erkenntnisse, insbesondere aus systematischen Übersichtsarbeiten, auch einem breiteren Publikum besser zugänglich zu machen, wurde die Datenbank „Wissen für gesunde Lebenswelten“ entwickelt, die seit dem 17.11.2020 online zugänglich ist [5]. Die Konzeption sowie die weitere Entwicklung der Datenbank erfolgen durch die BZgA im Auftrag des GKV-Spitzenverbandes gemäß §20a Abs. 3 und 4 SGB V.

Ziel dieses Beitrags ist es, diese neue Datenbank vorzustellen. Zunächst werden Ziele der Datenbank und das methodische Vorgehen bei der Erstellung der Datenbankeinträge beschrieben. Anschließend folgt eine Darstellung aktueller Datenbankinhalte sowie der Nutzungsmöglichkeiten für Krankenkassen und weitere Fachkräfte und Beteiligte in der Praxis. Wir erläutern zudem die Entwicklung eines innovativen Instruments zur Bewertung der methodischen Qualität von Übersichtsarbeiten. Die Diskussion gibt schließlich einen Ausblick auf geplante Weiterentwicklungsschritte sowie auf die Einbindung der Datenbank in andere Maßnahmen zum Wissenschafts-Praxis-Transfer.

## Ziele der Datenbank „Wissen für gesunde Lebenswelten“

Die Datenbank soll Krankenkassen, für die Lebenswelten Verantwortliche wie Einrichtungsträger, Kommunen und weitere in der Praxis Tätige bei Entscheidungen zur Planung und Förderung von lebensweltbezogenen Maßnahmen der Gesundheitsförderung und Prävention unterstützen. Sie stellt hierfür wissenschaftliche Erkenntnisse zur Wirksamkeit von Interventionsansätzen sowie zum Erfolg von Umsetzungsstrategien verständlich und nutzerfreundlich dar. Grundlage der Datenbankeinträge sind verschiedene Formen systematischer Übersichtsarbeiten.

Systematische Übersichtsarbeiten identifizieren, bewerten und synthetisieren bestehende wissenschaftliche Erkenntnisse zu einer Forschungsfrage. Somit geben sie einen Überblick über ein Forschungsfeld und liefern Hinweise, ob und inwiefern ein bestimmter Interventionsansatz oder eine Umsetzungsstrategie grundsätzlich wirkt. Zunehmend enthalten systematische Übersichtsarbeiten auch Erkenntnisse zu Bedingungen und Kontextfaktoren, die zum Erfolg einer Intervention oder Umsetzungsstrategie beitragen [6, 7]. Diese Erkenntnisse können in der Praxis Tätige und Entscheidungstragende zur Beantwortung strategischer Planungsfragen nutzen, wie beispielsweise der Frage, welche Interventionsansätze grundsätzlich geeignet sind, um eine spezifische Zielgruppe zu erreichen. Wenn klar ist, welcher Ansatz erfolgversprechend ist, können geeignete Maßnahmen der Gesundheitsförderung und Prävention gezielter recherchiert und eingesetzt werden. So kann eine Datenbank mit Erkenntnissen aus systematischen Übersichtsarbeiten bestehende Interventionsdatenbanken sinnvoll ergänzen.

Aktuell in Deutschland verfügbar sind Interventionsdatenbanken mit unterschiedlichen Schwerpunkten, die Erkenntnisse zu jeweils spezifischen Interventionen und Good-Practice-Beispielen im Bereich der Gesundheitsförderung und Prävention strukturiert zusammenfassen. Hierzu gehören unter anderem die „Grüne Liste Prävention“ [8], die Praxisdatenbank des Kooperationsverbundes „Gesundheitliche Chancengleichheit“ [9] sowie die Projektdatenbank „Gesund und aktiv älter werden“ [10]. Zusätzliche Interventionsdatenbanken auf Ebene einzelner Bundesländer ergänzen diese. Hierzu zählen zum Beispiel die Projektdatenbanken des Landeszentrums für Gesundheit Nordrhein-Westfalen [11], des Netzwerks Prävention in Bayern [12] sowie der Sächsischen Landesvereinigung für Gesundheit [13].

Eine Datenbank, die Erkenntnisse aus systematischen Übersichtsarbeiten zu lebensweltbezogenen Maßnahmen der Gesundheitsförderung und Prävention zusammenfasst, gibt es in Deutschland nach Kenntnisstand der Autorinnen und Autoren bislang noch nicht. International sind übersichtsarbeitbasierte Datenbanken bereits etabliert. Eine der bekanntesten ist die Datenbank der Cochrane Collaboration, die auch ausschließlich nach systematischen Reviews der „Public Health Group“ durchsucht werden kann. Für einen Großteil der systematischen Übersichtsarbeiten in der „Cochrane Library“ sind mehrsprachige Zusammenfassungen in einfacher Sprache öffentlich abrufbar [14]. Die kanadische Datenbank „Health Evidence“ der McMaster-Universität und des National Collaborating Centre for Methods and Tools stellt seit dem Jahr 2005 Erkenntnisse aus systematischen Übersichtsarbeiten zu Public-Health-Maßnahmen zur Verfügung. „Health Evidence“ richtet sich an Entscheidungstragende vor Ort sowie Wissenschaftlerinnen und Wissenschaftler im Bereich Public Health und Gesundheitsförderung. Daten zum Nutzungsverhalten belegen, dass die Datenbank stark frequentiert wird und die anvisierten Nutzergruppen tatsächlich erreicht [7]. In der Datenbank „Health Evidence“ wird eine vermehrte Recherche von Übersichtarbeiten zu Themen aktueller politischer Prioritäten beobachtet. Dies deutet darauf hin, dass „Health Evidence“ für evidenzinformierte Entscheidungen herangezogen wird [15].

Ziel der Datenbank „Wissen für gesunde Lebenswelten“ ist es, auch in Deutschland eine Struktur zu etablieren, welche die Wissensbasis zu Wirksamkeit und Nutzen von Gesundheitsförderungs- und Präventionsansätzen transparent macht. Die Datenbank schließt dabei neben systematischen Reviews auch Rapid-Reviews und Scoping-Reviews ein und konzentriert sich auf lebensweltbezogene Maßnahmen der Gesundheitsförderung und Prävention, insbesondere für vulnerable Zielgruppen, wie zum Beispiel Menschen mit Behinderung oder ältere Menschen.

## Methodisches Vorgehen bei der Erstellung der Datenbankeinträge

Bei der Erstellung der Datenbankeinträge standen folgende Überlegungen im Mittelpunkt: Die Erstellung sollte qualitätsgesichert erfolgen und die Einträge sollten sowohl in Bezug auf Inhalt als auch Form niedrigschwellig zugänglich sein sowie sich an den Bedarfen der anvisierten Nutzerinnen und Nutzer orientieren.

Für die qualitätsgesicherte Erstellung der Datenbankeinträge wurde ein standardisiertes Formular entwickelt, welches die erforderlichen Inhalte der Datenbank abbildet. Zwei Personen mit Expertise in Bezug auf systematische Übersichtsarbeiten fassen die zentralen Informationen aus jeder Übersichtsarbeit unabhängig voneinander in diesem Formular zusammen. Die beiden Versionen werden anschließend verglichen, Unterschiede diskutiert und eine finale Version abgestimmt. Dieses Verfahren entspricht etablierten Standards für die Erstellung von Evidenzsynthesen, die zur Fehlervermeidung und zur Förderung sicherer Entscheidungen die Anwendung eines standardisierten Extraktionsformulars und des Vieraugenprinzips empfehlen [16, 17]. Die an der Erstellung der Datenbankeinträge beteiligten externen Expertinnen und Experten sind von der BZgA beauftragt. Sie sind an unterschiedlichen wissenschaftlichen Institutionen tätig.

Verschiedene Studien belegen die Relevanz niedrigschwellig zugänglicher Informationen. So sind komprimierte Zusammenfassungen von Erkenntnissen aus Übersichtsarbeiten für Fachkräfte und Beteiligte in der Praxis verständlicher als Volltexte [18]. In der Praxis Tätige und Entscheidungstragende wünschen sich einen schnellen Überblick über zentrale Ergebnisse [19, 20]. Die Verwendung von Fachjargon und statistischen Begriffen stellt hingegen eine Barriere für die Nutzung von Erkenntnissen aus systematischen Übersichtsarbeiten dar [19]. In der Datenbank „Wissen für gesunde Lebenswelten“ werden Informationen entsprechend kurz dargestellt und Fachbegriffe erläutert. Die von Expertinnen und Experten erstellten Texte wurden anschließend von einer Wissenschaftsjournalistin sprachlich vereinfacht.

Mit dem Ziel, eine hohe Nutzerorientierung zu erreichen, wurden während des gesamten Entwicklungsprozesses der Datenbank Expertinnen und Experten der gesetzlichen Krankenkassen eingebunden. Die inhaltliche und technische Gestaltung der Datenbank wurde seit dem Jahr 2017 kontinuierlich in der Arbeitsgruppe (AG) Forschung, einem Gremium des GKV-Bündnisses für Gesundheit beraten. Sowohl die technische Grundkonzeption sowie die fertig umgesetzte Datenbank wurden von den Gremienmitgliedern getestet und auf Basis ihrer Rückmeldungen (u. a. zu verwendeten Suchfiltern, der Verschlagwortung sowie Platzierung der Suchfelder) angepasst. Die AG-Mitglieder haben dabei punktuell weitere Kolleginnen und Kollegen aus ihren jeweiligen Krankenkassensystemen einbezogen. Ende 2019 erfolgte zudem eine Testung der Datenbank durch potenzielle Nutzerinnen und Nutzer auf Ebene der Bundes- und Landesverbände der GKV sowie der Programmbüros des GKV-Bündnisses für Gesundheit. Die Testung wurde in einer geschützten Testumgebung durchgeführt. Rückmeldungen (*n* = 21) erfolgten per E‑Mail mittels eines semistrukturierten Fragebogens. Auf Basis der Rückmeldungen wurden Änderungen umgesetzt, u. a. in Bezug auf Bezeichnungen von Datenbankfeldern, die Zusammenführung von Feldern sowie zu vorgegebenen Auswahlkategorien. Dadurch sollte eine bessere Passung der Datenbankinhalte für den spezifischen Entscheidungskontext erzielt werden, um den Transfer in die Praxis zu fördern [20, 21].

## Inhalte und Nutzungsmöglichkeiten der Datenbank

Aktuell sind Einträge zu 13 Übersichtsarbeiten in der Datenbank enthalten, die im Auftrag des GKV-Bündnisses für Gesundheit erstellt wurden und insbesondere Erkenntnisse hinsichtlich wirksamer Strategien zur Verringerung sozial bedingter Ungleichheit von Gesundheitschancen generieren sollten. Die Auswahl der vulnerablen Zielgruppen und Fragestellungen, zu denen diese Übersichtsarbeiten erstellt wurden, erfolgte durch den GKV-Spitzenverband im Austausch mit der BZgA und den Verbänden der Krankenkassen auf Bundesebene.

Neben Fragestellungen zur Wirksamkeit von Maßnahmen für die ausgewählten vulnerablen Zielgruppen waren auch übergreifende Fragestellungen zu Konzepten, Theorien und Wissenslücken in verschiedenen Themenfeldern von Interesse. Zur Beantwortung dieser Fragestellungen wurden Scoping-Reviews beauftragt, die eine Übersicht zu bestehender Evidenz in einem Themenfeld im Sinne einer Evidence Map geben [22]. Für einige Fragestellungen war es notwendig, in kurzer Zeit eine Evidenzsynthese zu erhalten. Deshalb wurden auch Rapid-Reviews beauftragt, die Verfahrensschritte eines systematischen Reviews in verkürzter Form durchführen [23]. Im Rahmen einiger Übersichtsarbeiten wurden zudem ergänzende Experteninterviews durchgeführt.

Derzeit befinden sich 2 Rapid-Reviews in der Datenbank, 10 Scoping-Reviews und ein systematischer Review. Die Mehrzahl der bisherigen Datenbankeinträge beschreiben Erkenntnisse mit Bezug zur Lebenswelt Kommune (*n* = 12) und Schule (*n* = 8) sowie für die Zielgruppen Jugendliche (*n* = 5), junge Erwachsene (*n* = 3) und Kinder (*n* = 3). Darüber hinaus sind insbesondere Erkenntnisse zu den Themen Gesundheitskompetenz (*n* = 9), Alkohol- und Tabakkonsum (jeweils *n* = 7) sowie Stressbewältigung (*n* = 6) enthalten.

Nutzerinnen und Nutzer der Datenbank „Wissen für gesunde Lebenswelten“ finden auf der Startseite ein Verzeichnis aller in der Datenbank enthaltenen systematischen Übersichtsarbeiten, gelistet nach Titel, Publikationsjahr und Autorinnen und Autoren.

**Figure 1:**
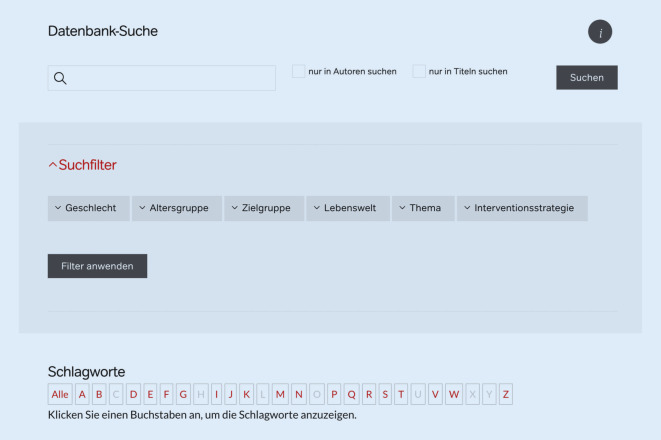

Zur Identifikation spezifischer Übersichtsarbeiten stehen auf der Startseite verschiedene Suchfunktionen zur Verfügung. Mit der Freitextsuche können Nutzerinnen und Nutzer in einem Suchfeld frei nach einem Begriff suchen. Sie können darüber hinaus angeben, ob der Begriff nur in den Feldern „Autoren“ oder den „Titeln“ der Übersichtsarbeiten gesucht werden soll. Mit der Schlagwortsuche können Nutzerinnen und Nutzer die Ergebnisse nach einzelnen Schlagworten filtern. Dies erfolgt mittels einer Schlagwortliste, aus der die Nutzerinnen und Nutzer zunächst die Anfangsbuchstaben und anschließend das gewünschte Schlagwort auswählen. Alternativ können die Ergebnisse auch durch Anwendung von Suchfiltern reduziert werden. Dafür wurden 6 Suchfilter angelegt, die eine Einschränkung der Suche nach Lebenswelt, Zielgruppe, Thema, Geschlecht, Altersgruppe und Interventionsstrategie ermöglichen. Datenbanknutzerinnen und -nutzer können mehrere Suchfilter gleichzeitig anwenden. Sie können die verschiedenen Suchfunktionen auch miteinander kombinieren, zum Beispiel indem sie erst über die Freitextsuche oder die Funktion Schlagwort suchen und die Ergebnisse anschließend über die Suchfilter weiter eingrenzen. Eine Erläuterung der Suchfunktionen ist über den Informationsbutton auf der Startseite zugänglich (Abb. 1, *oben rechts*).

**Figure 2:**
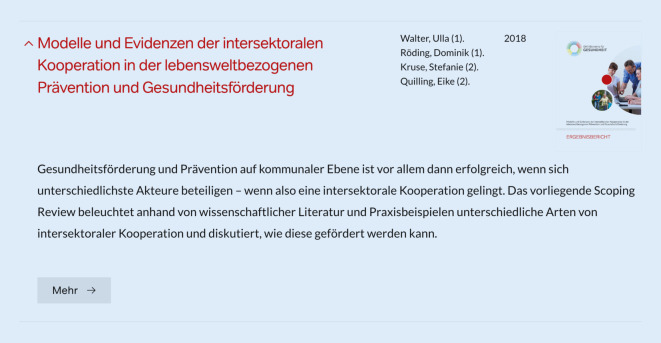

Die Inhalte jeder Übersichtsarbeit sind in 3 unterschiedlichen Detailtiefen dargestellt. Jeder Datenbankeintrag besteht aus einem Teasertext, einem Abstract sowie einer ausführlicheren Zusammenfassung. Der Teasertext erscheint nach Anklicken des Titels der Übersichtsarbeit auf der Startseite. Er liefert eine Kurzinformation zum Inhalt der Übersichtsarbeit (s. Beispiel in Abb. 2).

**Figure 3:**
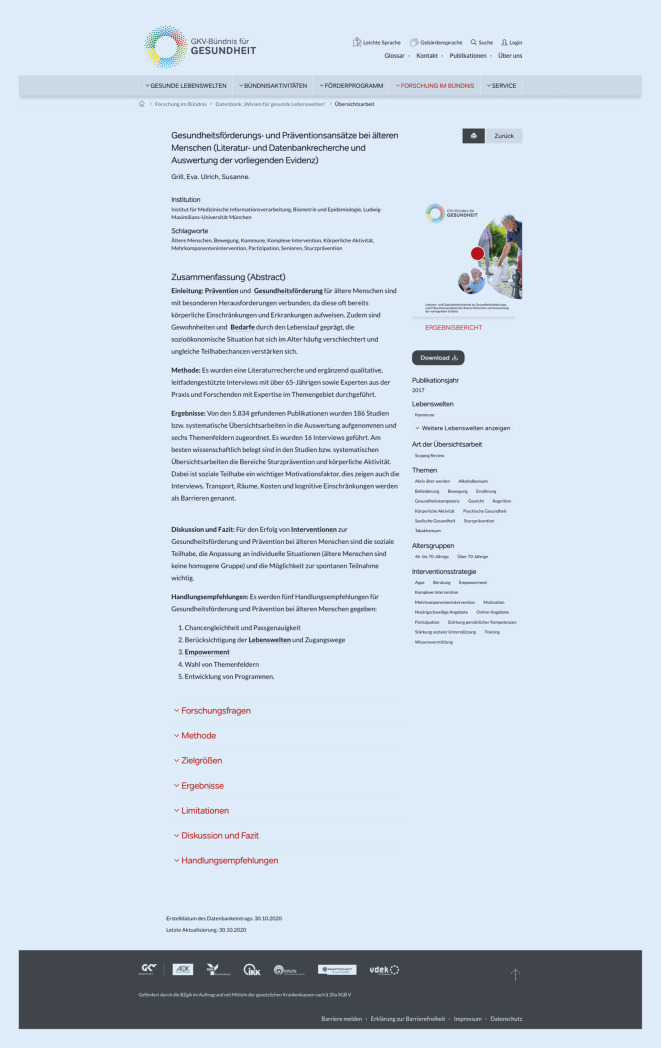

Nach Anklicken des Begriffs „Mehr“ wird der Abstract angezeigt (Abb. 3). Darunter befindet sich die ausführliche Zusammenfassung. Diese beschreibt in separaten Feldern die beantworteten Forschungsfragen, verwendeten Methoden, Zielgrößen, Kernergebnisse, Limitationen der Übersichtsarbeit sowie die wichtigsten Diskussionspunkte mit einem Fazit. Sofern möglich, werden zudem konkrete Handlungsempfehlungen dargestellt. Die Datenbankfelder „Limitationen“, „Handlungsempfehlungen“ und „Diskussion und Fazit“ können auch Einschätzungen der Expertinnen und Experten beinhalten, die den Datenbankeintrag erstellt haben. Daher können die Inhalte von den entsprechenden Abschnitten in der Originalarbeit abweichen. Um das Lesen zu erleichtern, kann jedes dieser Felder bei Bedarf auf- und zugeklappt werden. Jeder Datenbankeintrag enthält auch einen Link zur Originalübersichtsarbeit.

## Qualitätsbewertung der Übersichtsarbeiten

Der praktische Nutzen von Zusammenfassungen von Übersichtsarbeiten kann durch die Ergänzung von Bewertungen ihrer methodischen Qualität erhöht werden. Diese Information kann in der Praxis Tätige dabei unterstützen, Erkenntnisse aus systematischen Übersichtsarbeiten besser einzuordnen und ihren Informationswert für mögliche Entscheidungen einzuschätzen. Dies ist insbesondere dann hilfreich, wenn zu einem Thema mehrere Übersichtsarbeiten mit unterschiedlichen Ergebnissen vorliegen [24, 25]. In der Datenbank „Wissen für gesunde Lebenswelten“ sollen daher in einer nächsten Entwicklungsphase auch Qualitätsbewertungen ergänzt werden. Das Leibniz-Institut für Präventionsforschung und Epidemiologie – BIPS hat für das GKV-Bündnis für Gesundheit ein Qualitätsbewertungsinstrument entwickelt. Das als „Critical Appraisal Tool for Health Promotion and Prevention Reviews“ (CAT HPPR) bezeichnete Instrument wurde unter Berücksichtigung der bisher vorliegenden Übersichtsarbeiten des GKV-Bündnisses für Gesundheit erarbeitet. Für die vertretenen Reviewformate wiesen vorhandene Bewertungsinstrumente eine unzureichende Passung auf, dies galt insbesondere für Rapid- und Scoping-Reviews. Folglich wurde ein innovatives Instrument entwickelt, welches auf neuen Arbeitsdefinitionen zentraler Reviewformate (inkl. ergänzender Ansätze) beruht und sich an bestehenden Bewertungsinstrumenten (u. a. healthevidence.org, AMSTAR 2 [A MeaSurement Tool to Assess Systematic Reviews]) orientiert [25, 26] Das CAT HPPR besteht aus 15 Bewertungskriterien, die aus etablierten Critical Appraisal Tools (CATs) wie healthevidence.org sowie 13 weiteren bestehenden CATs extrahiert und adaptiert wurden. Die Auswahl dieser Kriterien erfolgte anhand ihrer objektiven Bewertbarkeit, Verständlichkeit und Relevanz. Bereiche der Bewertung, die bislang in CATs unberücksichtigt waren, wurden auf Basis der Anforderungen aktueller, internationaler Berichtsleitlinien und wissenschaftlicher Erkenntnisse ergänzt. Die Gesamtbewertung der Qualität einer Übersichtsarbeit erfolgt abhängig vom jeweiligen Reviewformat (systematischer, Scoping- oder Rapid-Review) und resultiert bei erfolgreicher Zuordnung zu einem Reviewformat in 4 möglichen Bewertungsstufen (hoch, moderat, niedrig, sehr niedrig). In Anlehnung an bestehende CATs wurde ein Ansatz gewählt, der unter anderem kritische Kriterien ausweist, die Übersichtsarbeiten erfüllen müssen, um nicht abgewertet zu werden. Im Rahmen des Entwicklungsprozesses des Instruments wurde ein Manual entwickelt und anhand der bereits vorliegenden Übersichtsarbeiten pilotiert. Die weitere Erprobung des CAT HPPR erfolgt im Zuge von Übersichtsarbeiten, die zukünftig in die Datenbank aufgenommen werden sollen.

## Diskussion

Die Datenbank „Wissen für gesunde Lebenswelten“ soll evidenzinformierte Entscheidungen bei Maßnahmen der lebensweltbezogenen Gesundheitsförderung und Prävention fördern. Eine internetbasierte Datenbank hat das Potenzial, viele für die Lebenswelten Entscheidungstragende auf effiziente Weise zu erreichen. Das GKV-Bündnis für Gesundheit ist bestrebt, die Datenbank auszubauen und als eine Informationsquelle zu etablieren, die Entscheidungstragende und in der Praxis Tätige bei Fragestellungen zu lebensweltbezogenen Maßnahmen der Gesundheitsförderung und Prävention als Erstes aufsuchen.

Zukünftig wird die Datenbank kontinuierlich um aktuelle Übersichtsarbeiten des GKV-Bündnisses für Gesundheit sowie aus weiteren Quellen ergänzt. Neben systematischen Reviews sollen weiterhin Scoping-Reviews und Rapid-Reviews berücksichtigt werden. In der Praxis Tätige und Entscheidungstragende haben erwiesenermaßen ein Interesse an unterschiedlichen Reviewtypen [21, 27]. Der Einschluss von Scoping-Reviews und Rapid-Reviews unterscheidet die Datenbank „Wissen für gesunde Lebenswelten“ auch von bestehenden Datenbanken und schließt somit eine Lücke. Neben der Ergänzung neuer Inhalte sind Befragungen der Nutzerinnen und Nutzer vorgesehen, deren Ergebnisse in die Weiterentwicklung der Datenbank einfließen werden. Möglichkeiten der Vernetzung der Datenbank mit bestehenden Interventionsdatenbanken (u. a. der Praxisdatenbank des Kooperationsverbundes „Gesundheitliche Chancengleichheit“) sollen zudem eruiert werden.

Für einen erfolgreichen Wissenschafts-Praxis-Transfer ist häufig eine Kombination verschiedener Strategien erforderlich [19, 28]. Daher ist geplant, die nutzergerechte Zusammenfassung systematischer Übersichtsarbeiten und deren Bereitstellung über die Datenbank mit weiteren Maßnahmen zum Wissenschafts-Praxis-Transfer zu begleiteten. Hierzu gehören Qualifizierungsangebote, die sich insbesondere an die für die Lebenswelten Verantwortlichen sowie weitere Fachkräfte und Beteiligte in der Praxis richten, u. a. solche zum systematischen Gesundheitsförderungsprozess und zu evidenzbasierten Strategien der kommunalen Gesundheitsförderung. Ergänzend zu Qualifizierungsmaßnahmen zur Befähigung zur evidenzinformierten Entscheidungsfindung empfehlen Studien für den erfolgreichen Praxistransfer auch den Austausch zwischen Akteurinnen und Akteuren aus Praxis und Wissenschaft [21]. Ein solcher Austausch findet bereits an verschiedenen Stellen im GKV-Bündnis für Gesundheit statt, u. a. in Form der Präsentation und Reflexion von Ergebnissen sowie der Durchführung von Expertinnen- und Experten-Hearings im Rahmen von Forschungsaufträgen. Der Austausch soll zukünftig unter Nutzung verschiedener Austauschformate ausgebaut werden.

Wissenschaftliche Erkenntnisse zum Praxistransfer systematischer Übersichtsarbeiten zeigen, dass in der Praxis Tätige und Entscheidungstragende einen Bedarf nach konkreten Handlungsempfehlungen haben, den systematische Übersichtsarbeiten üblicherweise nicht erfüllen können [19, 20]. Das GKV-Bündnis für Gesundheit unterstützt daher auch die Reflexion von Ergebnissen mit Krankenkassen und weiteren in der Praxis Tätigen sowie die Entwicklung von Handlungsempfehlungen zu verschiedenen Fragestellungen. So wurde z. B. zur Erstellung eines Handlungsrahmens für eine Beteiligung der gesetzlichen Krankenkassen mit Präventionsmaßnahmen nach §20a SGB V an multiprofessionellen und rechtskreisübergreifenden Hilfssystemen für Kinder und Jugendliche psychisch oder suchtkranker Eltern im Januar 2021 ein Reflexionsworkshop durchgeführt. Unter wissenschaftlicher Leitung nahmen daran sowohl Vertreterinnen und Vertreter von Krankenkassen, Krankenkassenverbänden und dem GKV-Spitzenverband als auch von Kommunen teil.

Für die Weiterentwicklung der Datenbank ist neben inhaltlichen Ergänzungen auch eine weitere Standardisierung und Optimierung des Verfahrens zur Erstellung der Datenbankeinträge vorgesehen. Derzeit werden verschiedene mögliche Quellen zur Ergänzung der Datenbank um weitere, nicht im Auftrag des GKV-Bündnisses für Gesundheit erstellte Übersichtsarbeiten geprüft. Die Standardisierung und Optimierung des Verfahrens zur Erstellung der Datenbankeinträge erfolgen fortlaufend anhand der Erfahrungen und Rückmeldungen der beteiligten Expertinnen und Experten.
