# Erratum zu: Wissen für gesunde Lebenswelten: Eine Datenbank zum Praxistransfer von Erkenntnissen aus systematischen Übersichtsarbeiten

**DOI:** 10.1007/s00103-021-03428-4

**Published:** 2021-10-05

**Authors:** Adrienne F. G Alayli, Christine Witte, Wolfgang Haß, Hajo Zeeb, Thomas L. Heise, Jens Hupfeld

**Affiliations:** 1 Abteilung 5 Unterstützung der Krankenkassen bei Leistungen zur Gesundheitsförderung und Prävention in Lebenswelten, Bundeszentrale für gesundheitliche Aufklärung (BZgA), Maarweg 149–161, 50825 Köln, Deutschland; 2 Referat Prävention, GKV-Spitzenverband, Berlin, Deutschland; 3 Leibniz-Institut für Präventionsforschung und Epidemiologie – BIPS, Bremen, Deutschland; 4 Health Sciences Bremen, Universität Bremen, Bremen, Deutschland

**Erratum zu:**

**Bundesgesundheitsbl 2021**

10.1007/s00103-021-03309-w

Der Artikel „Wissen für gesunde Lebenswelten: Eine Datenbank zum Praxistransfer von Erkenntnissen aus systematischen Übersichtsarbeiten“ von Adrienne F. G Alayli, Christine Witte, Wolfgang Haß, Hajo Zeeb, Thomas L. Heise und Jens Hupfeld wurde ursprünglich Online First ohne Open Access auf der Internetplattform des Verlags publiziert. Nach der Veröffentlichung in Bd. 64, Heft 5, pp. 552–559 hatten sich die Autoren für eine Open-Access-Veröffentlichung entschieden. Das Urheberrecht des Artikels wurde deshalb in © The Author(s) 2021 geändert. Dieser Artikel ist jetzt unter der Creative-Commons-Namensnennung 4.0 International-Lizenz veröffentlicht, welche die Nutzung, Vervielfältigung, Bearbeitung, Verbreitung und Wiedergabe in jeglichem Medium und Format erlaubt, sofern Sie den/die ursprünglichen Autor(en) und die Quelle ordnungsgemäß nennen, einen Link zur Creative-Commons-Lizenz beifügen und angeben, ob Änderungen vorgenommen wurden.

Die in diesem Artikel enthaltenen Bilder und sonstiges Drittmaterial unterliegen ebenfalls der genannten Creative-Commons-Lizenz, sofern sich aus der Abbildungslegende nichts anderes ergibt. Sofern das betreffende Material nicht unter der genannten Creative-Commons-Lizenz steht und die betreffende Handlung nicht nach gesetzlichen Vorschriften erlaubt ist, ist für die oben aufgeführten Weiterverwendungen des Materials die Einwilligung des jeweiligen Rechteinhabers einzuholen.

Weitere Details zur Lizenz entnehmen Sie bitte der Lizenzinformation auf http://creativecommons.org/licenses/by/4.0/deed.de.

